# Delays in the decision to seek care and associated factors among women in Ethiopia: systematic review and meta-analysis

**DOI:** 10.1186/s12884-025-07968-4

**Published:** 2025-08-11

**Authors:** Tesfahun Simon Hadaro, Misgun Shewangizaw, Teketel Ermias Geltore, Merkin Bekele, Lakew Desta Zewude, Mesfin Abebe, Yilma Chisha, Kusse Urmale Mare, Simeon Meskele Leyto

**Affiliations:** 1 Department of Midwifery, College of Medicine and Health Sciences, Arba Minch University, Arba Minch, Ethiopia; 2 School of Public Health, College of Medicine and Health Sciences, Arba Minch University, Arba Minch, Ethiopia; 3 Department of Midwifery, College of Health and Medical Sciences, Wachemo University, Hossana, Ethiopia; 4 Department of Midwifery, College of Medicine and Health Sciences, Dilla University, Dilla, Ethiopia; 5 Department of Nursing, College of Medicine and Health Sciences, Samara University, Samara, Ethiopia; 6 Department of Biomedical Sciences, College of Medicine and Health Sciences, Wachemo University, Hossana, Ethiopia

**Keywords:** Delay in seeking, Institutional delivery service, Emergency obstetric care, Women, Ethiopia

## Abstract

**Background:**

Delays in seeking care during pregnancy and childbirth can result in serious complications for women and a major contributor to maternal illness and death, especially in low-resource countries like Ethiopia. However, there is an inconsistency in findings of a study that has been conducted on the delay in decision to seek care. Thus, this review aimed to determine the combined prevalence of delay in decision to seek care and associated factors in Ethiopia.

**Methods:**

A systematic review and meta-analysis were conducted on the prevalence of delay in the decision to seek care and its associated factors. Five bibliographic databases and libraries, namely, Medline/PubMed, the Cochrane Library, Science Direct, Google Scholar, and Hinari, were used. Data extraction was performed using Microsoft Excel, and after cleaning and sorting, analysis was performed using STATA 17 software. The quality of each article was assessed using the Joanna Briggs Institute critical appraisal tool for prevalence studies. The pooled rate of delays in the decision to seek care and associated factors among mothers was estimated with a random-effects model. Heterogeneity was examined using Cochrane Q statistics and the I2 test, and publication bias was assessed by funnel plots and Egger’s regression test.

**Results:**

A total of twelve studies were included in this review. The pooled prevalence of delays in the decision to seek care at health facilities among women in Ethiopia was 44.69% (95% CI: 37.02–52.36). Several factors were significantly associated with these delays, including having no formal education (AOR = 4.48; 95% CI: 3.30–5.60), residing in rural areas (AOR = 5.40; 95% CI: 2.30–8.50), being unemployed (AOR = 2.60; 95% CI: 1.80–3.49), husband-only decision-making (AOR = 2.80; 95% CI: 1.13–4.50), and lack of antenatal care follow-up (AOR = 2.90; 95% CI: 1.30–4.10).

**Conclusion:**

The high pooled prevalence of delayed decision-making in seeking care highlights the urgent need for targeted interventions. These should focus on enhancing access to healthcare services, increasing maternal education, and promoting consistent antenatal care follow-up. Addressing these areas can significantly reduce delays and lead to improve maternal and public health outcomes.

**Supplementary Information:**

The online version contains supplementary material available at 10.1186/s12884-025-07968-4.

## Introduction

Each day, approximately 810 mothers lose their lives worldwide due to complications related to pregnancy or childbirth [1]. Sub-Saharan Africa carries a significant burden of maternal mortality, accounting for 86%. Alarmingly, the majority of these fatalities occur during labor or in the immediate postpartum period, highlighting the urgent need for improved maternal healthcare in this region [1, 2].

Thaddeus and Maine’s three-phase model, formulated in 1994, provides a framework for identifying indirect factors contributing to maternal mortality. This model emphasizes three key stages: the initial delay, marked by hesitation in deciding to seek care in a healthcare facility (a significant factor contributing to the elevated rates of maternal mortality in developing countries); the second delay, linked to identifying and accessing healthcare facilities; and the third delay, which includes delays in receiving adequate care at the healthcare facility [3].

Delays in deciding to seek care during pregnancy and childbirth can significantly increase the risk of serious complications. Conditions like severe bleeding, obstructed labor, premature rupture of membranes, and obstetric fistulas are among the leading causes of maternal death [4].

Various factors contribute to maternal health complications resulting from a lack of proactive decision-making when seeking medical care in health facilities. These factors are intricately linked to inadequate access to antenatal care, age disparities, educational attainment, and employment status [5]. In addition, other study findings highlight numerous factors at both individual and household levels showed challenges in accessing appropriate obstetric care, including transportation limitations, financial constraints, younger age, illiteracy, lower income, unemployment, limited utilization of health services, unplanned pregnancy, husband’s educational status, inadequate awareness of obstetric danger signs, and cultural beliefs [6, 7].

Differences in the first delay in seeking care were noted among women in Ethiopia across various regions [8–15]. Despite ongoing efforts to strengthen health extension programs and expand the coverage of maternal health services, timely access, especially in terms of reducing delays in the decision to seek care, remains a significant challenge [16]. Although individual studies have investigated maternal delays and associated factors among mothers seeking maternal care in various regions [8–15]. This systematic review and meta-analysis aimed to estimate the pooled prevalence of delays in the decision to seek care and identify associated factors among mothers in Ethiopia, addressing the lack of comprehensive and representative data.

## Methods

We adhered to the Preferred Reporting Items for Systematic Reviews and Meta-Analyses (PRISMA) guidelines and registered our protocol with PROSPERO (registration number: CRD42024527973) to maintain transparency and consistency throughout the review process.

### The eligibility criteria

#### Inclusion and exclusion criteria

This systematic review and meta-analysis included articles that examined maternal delays in seeking care and associated factors in Ethiopia, specifically observational studies (cross-sectional, cohort, and case‒control designs) published in the English language between 2013 and 2024. Studies that did not address maternal delay in seeking care and were conducted outside Ethiopia were excluded.

### Search strategy

We searched from the following databases: Midline/PubMed, Cochrane Library, Science Direct, Google Scholar, and Hinari. The search followed the CoCoPop framework (Condition (Co): Delay in making the decision to seek maternal healthcare services, specifically related to care during pregnancy, labor and delivery, or the postpartum period. Context (Co): Studies carried out in Ethiopia, covering both rural and urban environments, and involving various levels of healthcare facilities, including health centers and hospitals. Population (Pop), postpartum mothers who have given birth, with an emphasis on those who encountered delays in deciding to seek institutional delivery care) algorithm. MeSH terms or keywords were used for online database searches, with search terms combined using the Boolean operators “OR” and “AND.” Keywords such as (“Maternal delay” OR “First maternal delay” OR “Decision to seek care”) AND (“Women”) AND (“Determinants” OR “Factors” OR “Associated factors”) AND (“Ethiopia”) were used Table 1.

**Table 1:** Search strategy of the studies in delays in the decision to seek care and associated factors among women in Ethiopia 2024

Database	Search Terms / Strategy	Filters/Restrictions
PubMed/MEDLINE	(“delay in seeking care” OR “first delay” OR “decision to seek care”) AND (“maternal health” OR “maternal healthcare utilization”) AND “Ethiopia” AND (“mothers” OR “women”)	English language, human studies, last 10 years
Cochrane Library	(“delay in seeking care” OR “decision to seek care”) AND “maternal health services” AND “Ethiopia”	
ScienceDirect	TITLE-ABS-KEY(“delay in seeking care” OR “first delay”) AND (“maternal health” AND “Ethiopia”)	
Google Scholar	“delay in seeking care” AND “maternal health” AND “Ethiopia”	
Hinari	(“maternal health services” AND “delay in decision to seek care” AND “Ethiopia”)	

### Selection of studies

The search results were imported into citation manager (EndNote version 21), and duplicates were excluded. Subsequently, the selection of the studies occurred in two stages. Initially, the title and abstract selection were performed. Next, a comprehensive review of the full texts was conducted based on eligibility criteria. In the first stage, studies that mentioned the prevalence and/or determinants were identified for full-text review through title and abstract screening, which was carried out independently by four reviewers. During the full-text review, any article deemed potentially eligible by either reviewer was included for further evaluation, with both reviewers independently examining them. In instances of disagreement where a consensus could not be reached between both reviewers, a third reviewer reviewed the articles and resolved the discrepancies.

### Data extraction and management

Data extraction from the included studies was carried out using a standardized data abstraction form developed in an Excel spreadsheet. The data extraction process involved capturing the following parameters: author’s name, year of publication, study location, sample size for each study, study population, study design, prevalence, and factors.

### Assessment of risk of bias and quality assessment

The quality of the included studies was assessed by two authors (SM and MB) using the Joanna Briggs Institute (JBI) quality assessment tool, which has been adapted for prevalence studies [17]. The tool consists of nine criteria, which we used to systematically assess each included study. Two independent reviewers evaluated the studies against these criteria, which cover aspects such as initial population, sample size adequacy, data collection methods, tools used for data collection, statistical analysis, and response rate adequacy. Studies meeting at least 70% of the criteria were considered to have low risk of bias and were included in the final analysis. If there were any scoring disagreements, the sources of the disagreement were discussed and settled by another author (MS).

### Data processing and analysis

For data analysis, the STATA version 17 statistical programs were used. Inverse variance (I2) and Cochran Q statistics were utilized to investigate the heterogeneity of the studies. A random effects model with a 95% confidence interval (CI) was used to compute the pooled prevalence.

### Outcome measurements

Maternal delay in seeking care is defined as taking one hour or more to decide to seek care at a health facility [12]. The primary objective of this study was to estimate the pooled prevalence of such delays, while the secondary objective was to identify factors associated with the delay in decision-making among women. Odds ratios for these factors were calculated from the primary studies.

## Results

### Characteristics of the included studies

**Fig. 1:**
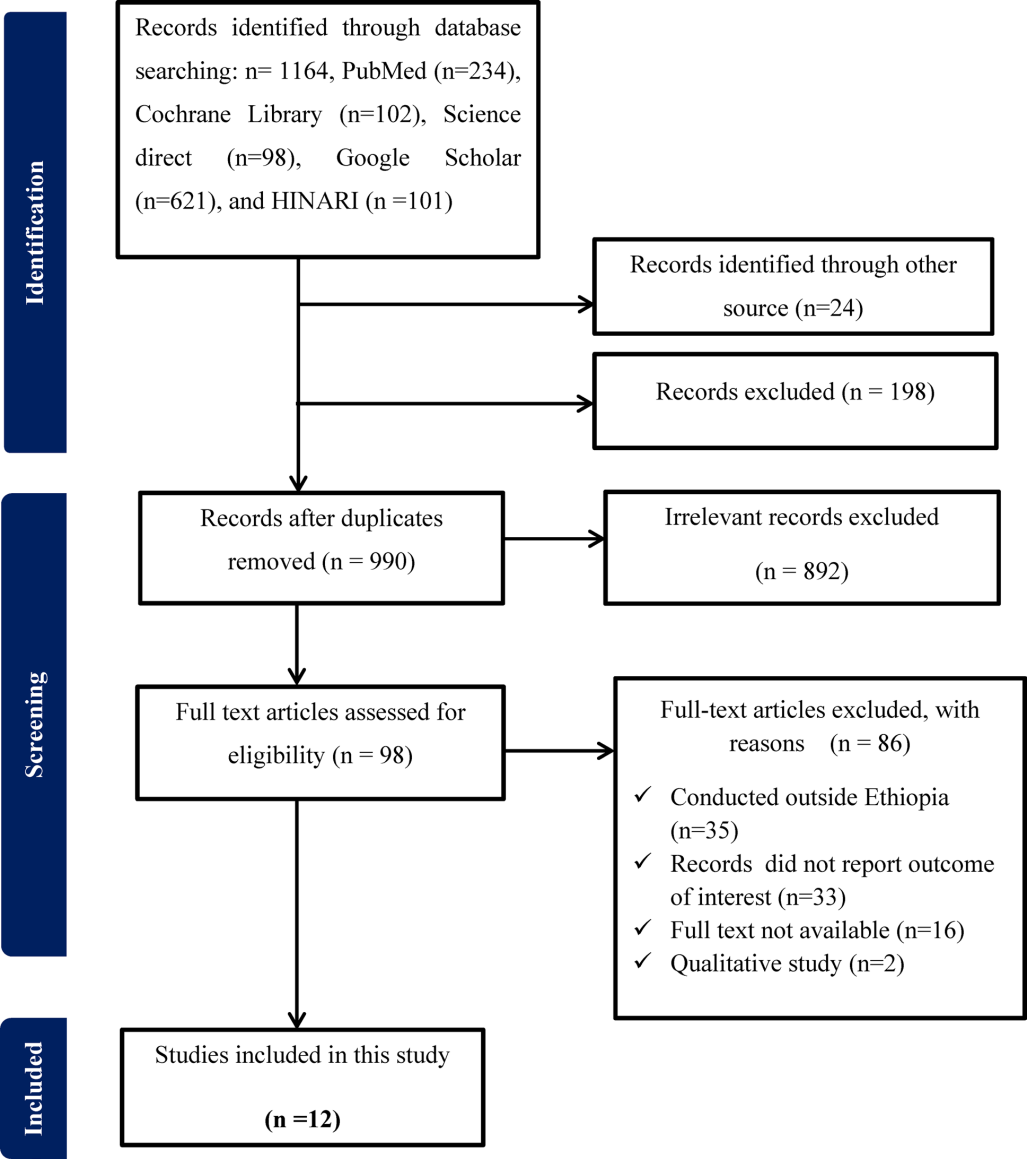
PRISMA flow chart for screening primary studies for systematic review and meta-analysis on the prevalence of delay in the decision to seek care and its associated factors among women in Ethiopia, 2024

A total of 1164 records were searched from the database, and 24 studies were included from other sources. After removing duplicates and excluding irrelevant titles during the title and abstract screening, ninety-eight articles were selected for full-text review. Following this, eighty-six were excluded with documented reasons, resulting in twelve studies being included. The main reasons for exclusion were conducted outside Ethiopia (*n* = 35), records don’t report outcome of interest (*n* = 33), full text not available (*n* = 16) and qualitative study (*n* = 2). Finally, 12 studies met the inclusion criteria in this systematic review and meta-analysis were included (Fig. 1).

This systematic review and meta-analysis included 12 facility-based cross-sectional studies, comprising a total of 5,268 participants. The sample sizes of the included studies ranged from 189 to 775. Regarding regional distribution, three of the studies were conducted in the Oromia Region [9, 11, 14], three in the Amhara region [10, 13], five in Southern Ethiopia [12, 15, 18–20], and one in Addis Ababa [8]. The quality of the included studies was assessed using the Joanna Briggs Institute tool. Studies with a JBI score of 70% and higher in the quality assessment scores were considered as low risk and included in the analysis shown in Table 2.

**Table 2:** The study characteristics included to identify the prevalence of delay in the decision to seek care and associated factors among women in ethiopia, 2024

Author	Region	Sample Size	Finding	Design	Setting	Quality
Endalkachew et al.(2020)	Addis Abeba	403	52.4	Cross-sectional	Facility	Low risk
Yirga et al. (2018)	Oromia	775	27.2	Cross-sectional	Facility	Low risk
Terefe (2019)	SNNPR	394	42	Cross-sectional	Facility	Low risk
Seifu et al (2020)	SNNPR	394	46.8	Cross-sectional	Facility	Low risk
Gebrehiwot et al(2021)	Amahara	459	59.7	Cross-sectional	Facility	Low risk
Dawit et al. (2020)	Amahara	650	36.3	Cross-sectional	facility	Low risk
Teklemariam (2018)	SNNPR	393	76.3	Cross-sectional	Facility	Low risk
Kelemu et al (2023)	SNNPR	410	42.1	Cross-sectional	Facility	Low risk
Derese et al. (2024)	Oromia	407	29.2	Cross-sectional	Facility	Low risk
Samira et al. (2021)	Oromia	189	46.7	Cross-sectional	Facility	Low risk
Alemu et al. (2017)	SNNPR	384	40.1	Cross-sectional	Facility	Low risk
Worku et al. (2013)	Amahara	410	37.8	Cross-sectional	Facility	Low risk

### Pooled prevalence of delays in the decision to seek care

**Fig. 2:**
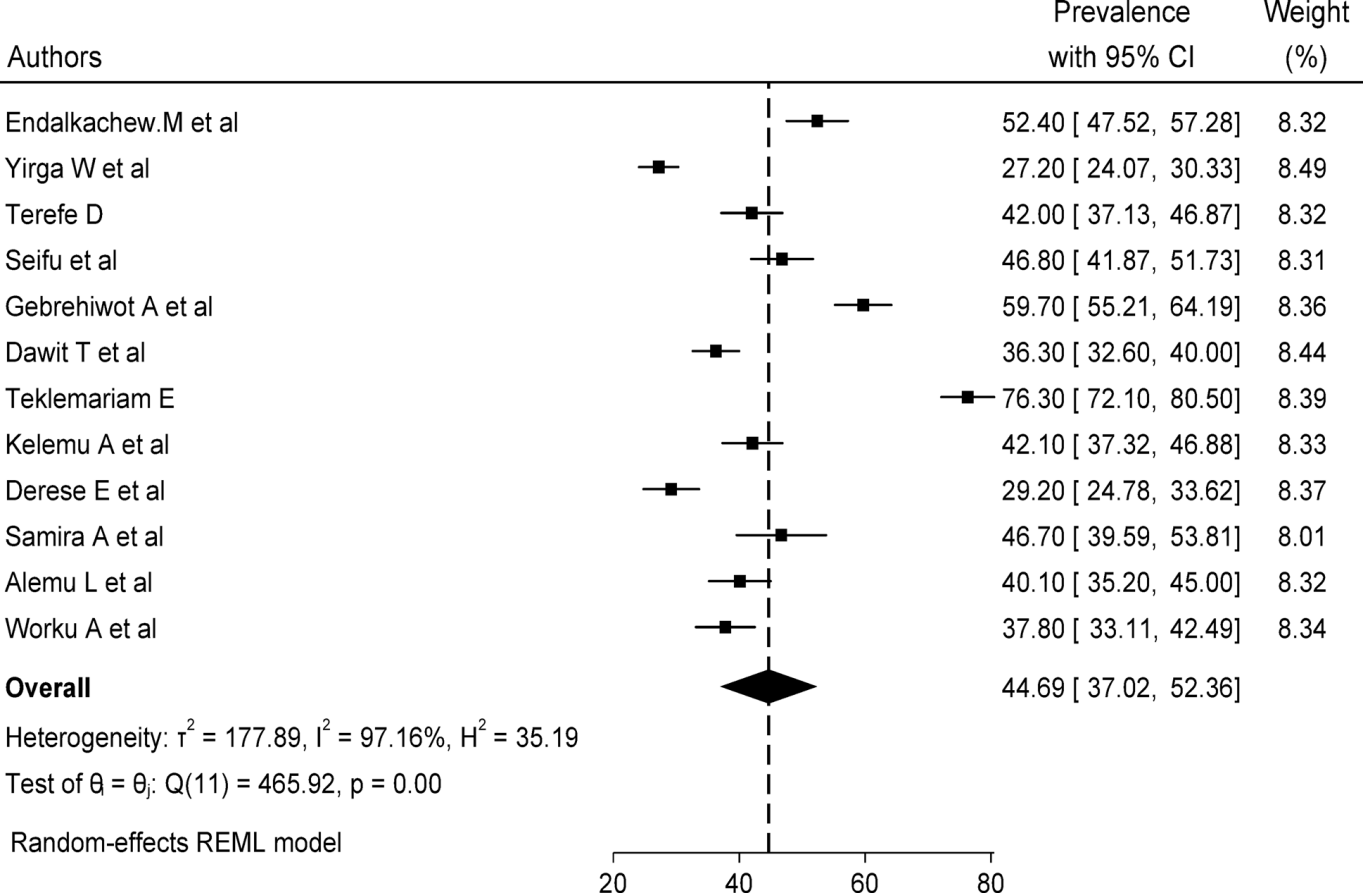
Pooled prevalence of delays in the decision to seek care in institutional services in Ethiopia, 2024

The pooled prevalence of delays in the decision to seek care in Ethiopia was estimated to be 44.69% (95% CI 37.02–52.36). Random effect analysis was performed due to the presence of significant heterogeneity between studies (I2 = 97.16%, *p* < 0.00). The lowest reported prevalence was 27.2%, and the highest reported prevalence was 76.3% (Fig. 2).

### Subgroup analysis

**Fig. 3:**
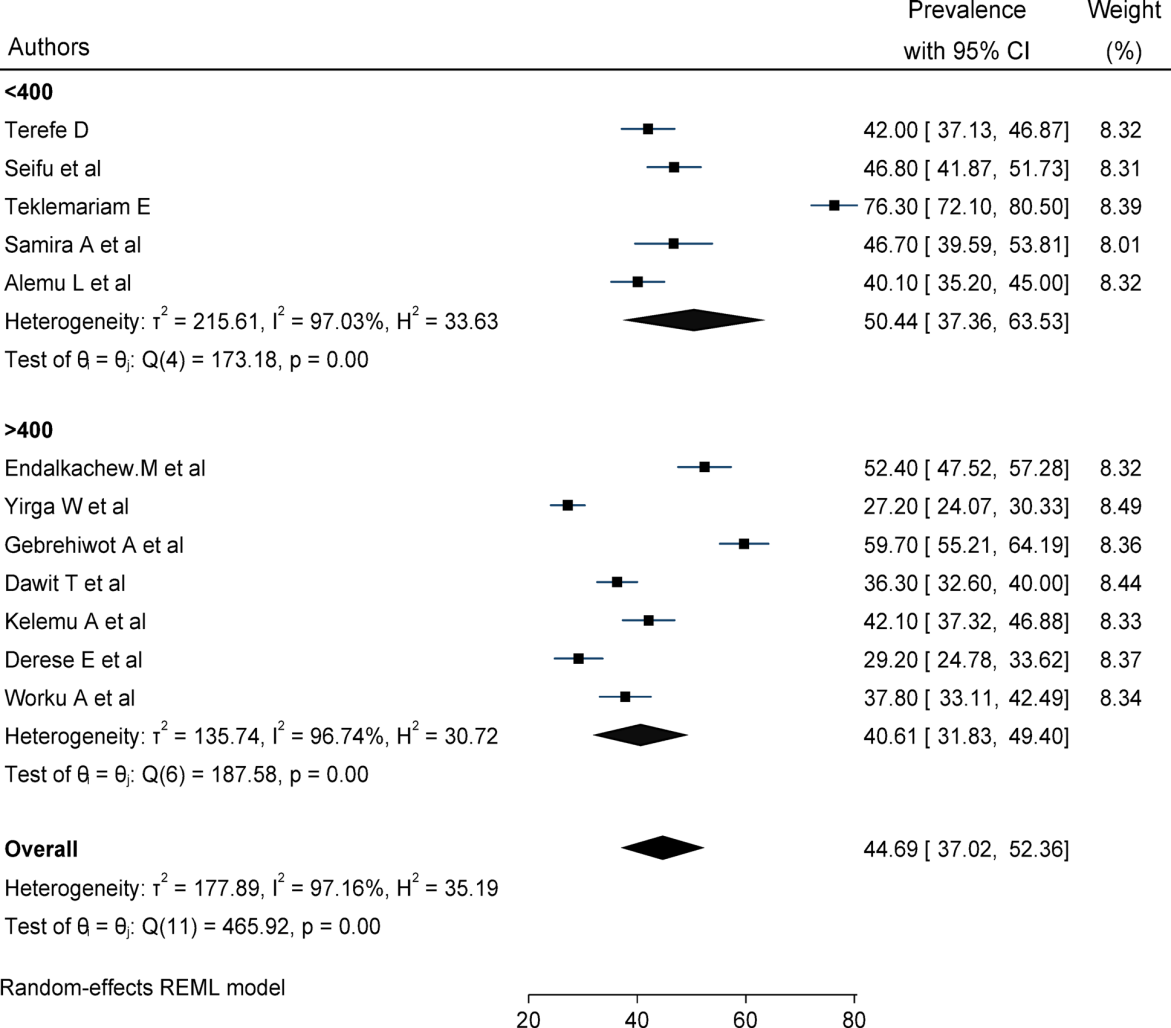
Subgroup analysis of the pooled prevalence of delays in the decision to seek care among women based on sample size in Ethiopia, 2024

**Fig. 4:**
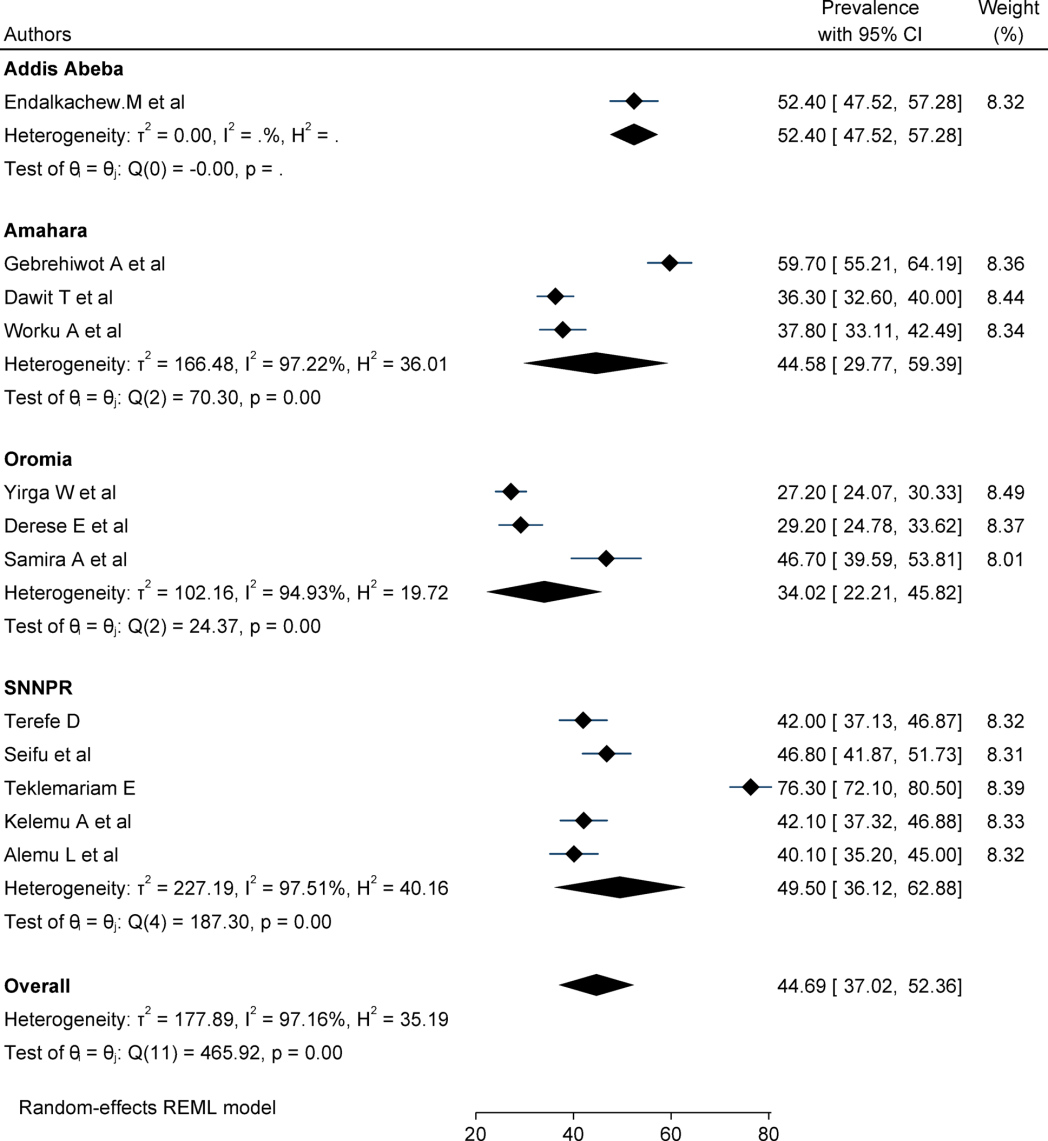
Subgroup analysis of the pooled prevalence of delays in the decision to seek care among women based on the region in Ethiopia, 2024

Due to significant heterogeneity between studies, subgroup analysis has been done based on region and sample size. Sub-group analysis among studies with a sample size less than 400 had the higher prevalence of delay in the decision to seek care (50.44%, CI 37.36–63.35), whereas a sample size greater than or equal to 400 had the lower prevalence (40.61%, CI: 31.8–49.4%) Fig. 3. The pooled prevalence of delays in the decision to seek care was higher in Addis Abeba [(52.4%: 95% CI: 47.5–57.2)], whereas the lowest prevalence observed in the Oromia region was 34.02% (95% CI: 22.2–45.8) Fig. 4.

### Sensitivity analysis

**Fig. 5:**
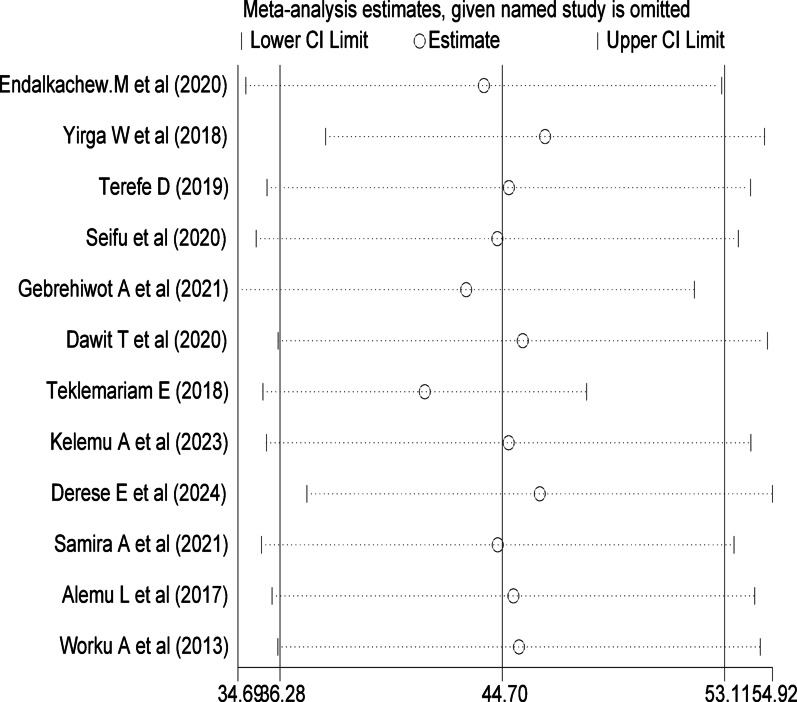
Sensitivity analysis of the delay in seeking care among Ethiopian women, 2024

We explored the sources of heterogeneity using a leave-one-out sensitivity analysis. The results indicated that removing any single study did not substantially affect the overall estimated prevalence. In each case, the prevalence remained within the 95% confidence interval of the pooled estimate, suggesting that no individual study had a disproportionate influence on the findings. The overall average prevalence remained stable at 44.69% (95% CI: 37.02–52.36) across all iterations (Fig. 5).

### Meta-regression

Substantial heterogeneity was observed between the studies. The observed heterogeneity among the included studies is likely due to differences in study settings, such as variations between rural and urban areas across Ethiopia, as well as inconsistencies in how delays in seeking care were measured ranging from self-reports to facility records. Additionally, variations in study designs, sample sizes, and data collection periods contributed to the diversity of findings.

To explore the sources of this heterogeneity, we conducted a meta-regression analysis, incorporating variables such as publication year and sample size. However, meta-regression analysis did not reveal a significant impact on the observed variation in delay of decision prevalence (Table 3).

**Table 3:** Shows meta-regression for delay in the decision to seek care among women in ethiopia, 2024

Source of heterogeneity	Coefficient	Standard error	*P* value
Sample size	-0.03	0.03	0.18
Publication year	0.17	1.6	0.91

### Publication biases

**Fig. 6:**
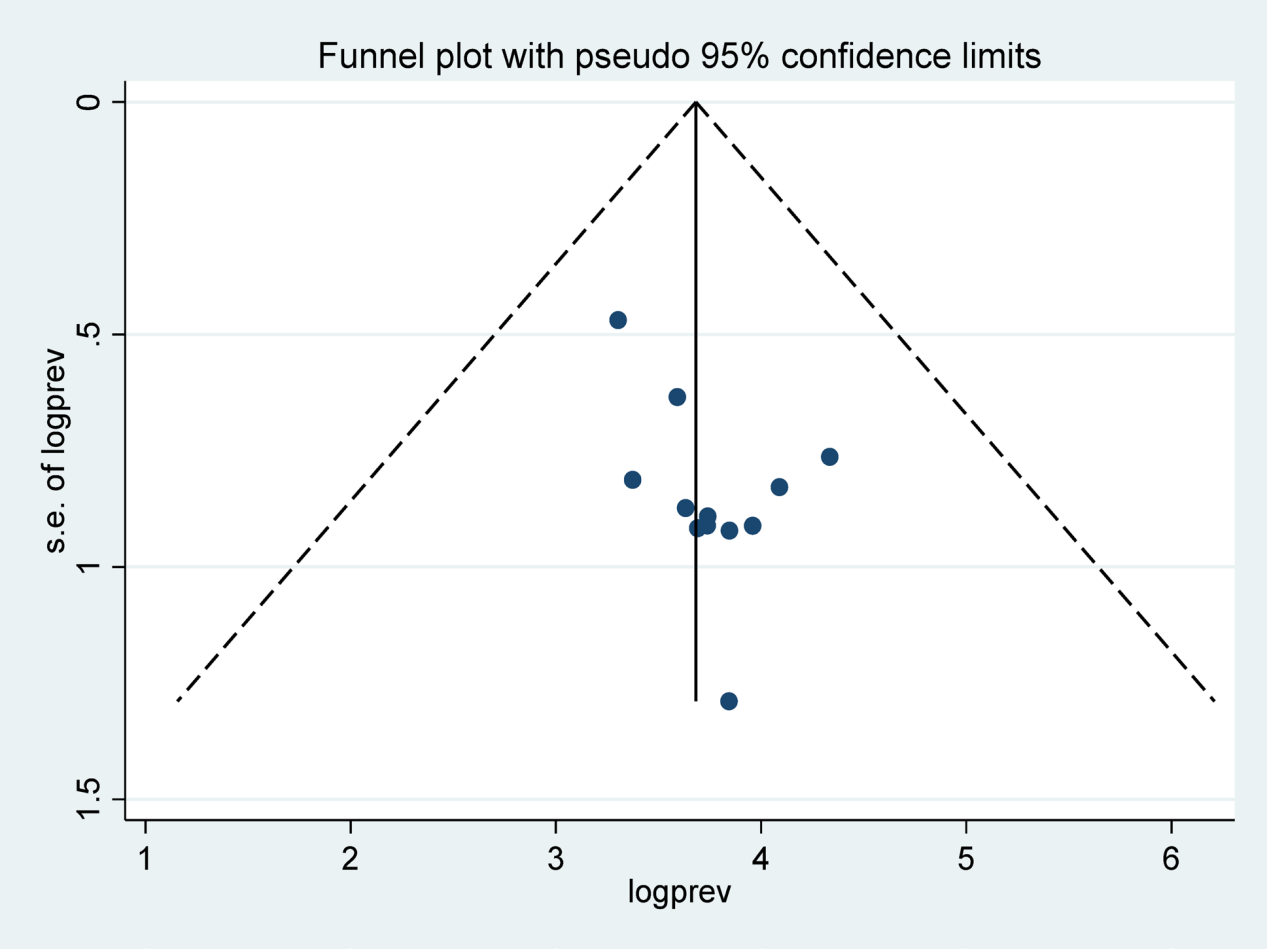
Funnel plot for assessing publication bias of the prevalence

To assess publication bias, we used Egger’s test alongside visually examining a funnel chart. Examination revealed symmetrical distribution in the selected studies with Eggers’ tests P value of 0.53, indicating there was no significant publication bias (Fig. 6).

### Factors associated with delays in decision to seek care in Ethiopia

This systematic review and meta-analysis identified key factors contributing to delays in seeking care among women in Ethiopia. These factors included no formal education, rural residence, maternal unemployment, lack of autonomy in decision-making (with husbands making decisions), and inadequate antenatal care follow-up Table 4.

Five studies identified a significant association between maternal education and delays in seeking care. Women with no formal education were 4.48 times more likely to delay care-seeking compared to educated women (AOR = 4.48; 95% CI: 3.30–5.60), with minimal heterogeneity (I² = 0.2%, *p* = 0.40). Three studies examined the role of residence. Women living in rural areas were 5.4 times more likely to delay seeking care than those in urban settings (AOR = 5.4; 95% CI: 2.3–8.5), with substantial heterogeneity across studies (I² = 86.2%, *p* = 0.00).

The association between unemployment and care-seeking delays was assessed in five studies. Unemployed women were 2.66 times more likely to delay seeking care than employed women (AOR = 2.66; 95% CI: 1.80–3.49), with no observed heterogeneity (I² = 0.0%, *p* = 0.68). Three studies explored household decision-making dynamics. Women whose husbands solely made healthcare decisions were 2.8 times more likely to delay seeking care (AOR = 2.8; 95% CI: 1.13–4.5).

Lack of antenatal care was also associated with delayed decision-making. According to three studies, women without ANC visits were 2.9 times more likely to delay seeking care than those who had ANC follow-up (AOR = 2.9; 95% CI: 0.9–4.1), with moderate heterogeneity (I² = 45.5%, *p* = 0.144).

**Table 4:** Factors associated with delays in the decision to seek care in ethiopia, 2024

Variable	Authors	AOR(95% CI)	Pooled AOR(95% CI)	Heterogenity
Maternal education (not formal education)	Gebrehiwot A et al.	4.6 (8.59–2.45)	4.47 (3.29–5.65)	I^2^ = 0.2%, *p* = 0.40
	Dawit T et al.	3.62(2.45–5.52)		
	Terefe D	6.79 (2.37–9.4)		
	Worku A et al.	6.71 (3.66–12.29)		
	Yirga W et al.	5.2(3.46–11.9)		
Residence (rural)	Terefe D	11.28 (5.94–14.27)	5.4 (2.3–8.5)	I^2^ = 86.2%, *p* = 0.00
	Gebrehiwot A et al.	4.1(2.36–6.25)		
	Dawit T et al.	3.14(2.4–4.01)		
Being an unemployed woman	Worku A et al.	3.94(2.36–6.57)	2.66 (1.80, 3.49)	I^2^ = 0.0%, *p* = 0.68
	Kelemu A et al.	2.52(1.5–4.13)		
	Samira A et al.	6(1.7–21.2)		
	Gebrehiwot A et al.	2.5(1.11–5.4)		
	Seifu et al.	2.2(1.15–4.16)		
No antenatal care follow-up	Terefe D	4.76(2.28–9.96)	2.9(0.9–4.1)	I^2^ = 45.5%, *p* = 0.14
	Yirga W et al.	4(3.3–9.1)		
	Gebrehiwot A et al.	1.8(1.32–3.180		
Husband decision	Worku A et al.	3.3(1.25–7.2)	2.8(1.13, 4.5)	I^2^ = 24.5%, *p* = 0.25
	Teklemariam E	2.08(1.09–3.95)		
	Seifu et al.	6(2.87–12.42)		

## Discussions

This systematic review and meta-analysis assessed the prevalence of delayed decisions to seek care and identified contributing factors among women in Ethiopia. The pooled prevalence was 44.69% (95% CI: 37.02–52.36), with considerable heterogeneity among studies. This figure is lower than that reported in Nigeria, where the prevalence reached (57%) [21], Pakistan (78%) [22], and Bangladesh (69.3%) [23]. This discrepancy may reflect differences in geographic setting, cultural norms, socio-demographic profiles, lifestyle, and the structure of healthcare systems influencing women’s access to and experiences with care.

However, the findings of this study were greater than those studies performed in Burundi (39.5%) [24] and Nepal (9.4%) [25]. Variations in the prevalence of delays in seeking care may stem from differences in how delay is defined and measured, as well as from diverse sample populations and cultural contexts that shape women’s perceptions of childbirth. Additionally, the prevalence may be influenced by the complex interplay of individual, social, psychological, and health system factors, which can vary significantly across countries.

This meta-analysis found that delays in seeking care were significantly associated with maternal education, residence, employment status, decision-making power, and antenatal care attendance. No formal education were more likely to delay seeking care than their counterparts, a finding consistent with research from Nepal [25, 26]. The possible reason might be that individuals with limited literacy may hinder individuals from understanding and responding to information about pregnancy risks, birth preparedness, and the use of institutional delivery services.

This study identified rural residence as a significant predictor of delayed care-seeking for institutional delivery. Mothers in rural areas were more than 5.4 times as likely to delay seeking care compared to those in urban settings. This may be due to limited decision-making autonomy, poor access to health facilities, inadequate transportation infrastructure, and insufficient health education on childbirth complications. In contrast, urban women often have better access to information through various media, making them more aware of the benefits of delivering in health facilities [27].

This study also found that unemployed mothers were significantly more likely to delay seeking care at health facilities compared to employed mothers. Specifically, unemployed women were 2.66 times more likely to delay care-seeking. This finding aligns with a study from the Jimma Zone, Ethiopia, which suggested that limited involvement in income-generating activities increases women’s financial dependence on their spouses. This dependency may hinder their autonomy and willingness to seek timely institutional delivery services [9].

The absence of antenatal care (ANC) follow-up was a significant factor contributing to delays in seeking care. This systematic review and meta-analysis revealed that women without ANC visits were 2.9 times more likely to delay the decision to seek care compared to those who attended at least one visit. A possible explanation is that ANC visits provide essential information about potential delivery complications, helping mothers to better prepare for childbirth and make timely decisions.

Women’s participation in decision-making plays a critical role in reducing delays in seeking healthcare and in the appropriate use of institutional delivery services, compared to decisions made solely by their husbands. This review found that when husbands were the sole decision-makers, the likelihood of delay was 2.8 times higher than when women made decisions independently. This finding is consistent with a study conducted in rural Bangladesh, which also highlighted the importance of women’s autonomy in promoting timely maternal healthcare utilization [23]. This could be attributed to the dominance of husbands or male partners in decision-making, which increases the risk of delay.

## Strengths and limitations of the study

Our systematic review strictly followed the PRISMA guidelines, utilized comprehensive search strategies, assessed the quality of included studies, and conducted a meta-analysis. However, this review has some limitations, including potential selective reporting due to the inclusion of only English-language publications and the exclusion of qualitative studies, which might have offered valuable insights into the experiences and perspectives of women facing delays in deciding to seek care.

## Conclusions

This review identified a high prevalence (44.69%) of delays in maternal decision-making to seek care in Ethiopia. Key contributing factors include lack of maternal education, rural residence, unemployment, limited decision-making power, and absence of antenatal care.

### Recommendations

Strengthen community-based maternal education programs targeting uneducated women. Expand maternal health services and outreach in rural areas. Promote women’s economic empowerment through access to income-generating opportunities. Support women’s autonomy in health-related decision-making within households. Enhance the coverage and quality of antenatal care services. Implement awareness campaigns on obstetric danger signs at the community and household levels.

### Implications of the review

The identified delays in mothers’ decisions to seek care in Ethiopia have critical implications for maternal and neonatal health. Addressing factors such as lack of maternal education, rural residence, unemployment, male-dominated decision-making, and absence of antenatal care is essential. Interventions should focus on empowering women through education, expanding access to maternal services in rural areas, promoting shared decision-making within households, and strengthening antenatal care utilization. Targeted strategies that tackle these barriers can significantly reduce delays and improve maternal and child health outcomes in Ethiopia.

## Supplementary Information

Below is the link to the electronic supplementary material.

Supplementary Material 1

## Data Availability

No datasets were generated or analysed during the current study.

## Abbreviations

AOR: Adjusted odds ratio
PRISMA: Preferred reporting items for systematic reviews and meta-analysis
PROSPERO: Prospective register of systematic reviews
SNNPR: Southern Nations, Nationalities, and Peoples’ Region
